# 4-[(3-Hy­droxy­anil­ino)­(phenyl)­methyl­idene]-3-methyl-1-phenyl-1*H*-pyrazol-5(4*H*)-one

**DOI:** 10.1107/S1600536812023082

**Published:** 2012-05-26

**Authors:** Keraghel Saida, Benghanem Fatiha, Dehbi Ouarda, Ourari Ali, Ouari Kamel, Lydia Brelot

**Affiliations:** a Laboratoire d’Electrochimie, d’Ingenierie Moléculaire et de Catalyse Redox, Departement de Génie des Procédés, Faculté de Technologie, Université Ferhat Abbas, Sétif, Algeria; b Institut de Chimie de Strasbourg, UMR 7177 CNRS-UdS, Service de Radiocristallographie, 1 rue Blaise Pascal, 67008 Strasbourg Cedex, France

## Abstract

In the title compound, C_23_H_19_N_3_O_2_, the dihedral angles formed by the pyrazolone ring with the three benzene rings are 30.91 (6), 60.96 (4) and 57.01 (4)°. The ligand is in the enamine–keto form and its structure is stabilized by an intra­molecular N—H⋯O hydrogen bond. In the crystal, O—H⋯N hydrogen bonds link mol­ecules into chains parallel to [01-1].

## Related literature

For the synthesis and applications of pyrazolo­nes and derivative compounds, see: Jensen (1959 ▶); Casas *et al.* (2007 ▶); Metwally *et al.* (1985 ▶); Morris *et al.* (1986 ▶); Raja *et al.* (2012 ▶); Delgado *et al.* (2006 ▶); Liskovskaya *et al.* (2006 ▶); Peng *et al.* (2004 ▶); Wang *et al.* (2002 ▶); Ramasamy *et al.* (2010 ▶); Thakar *et al.* (2010 ▶); Xu *et al.* (2006 ▶); Zhu *et al.* (2005 ▶); Wang *et al.* (2003 ▶).
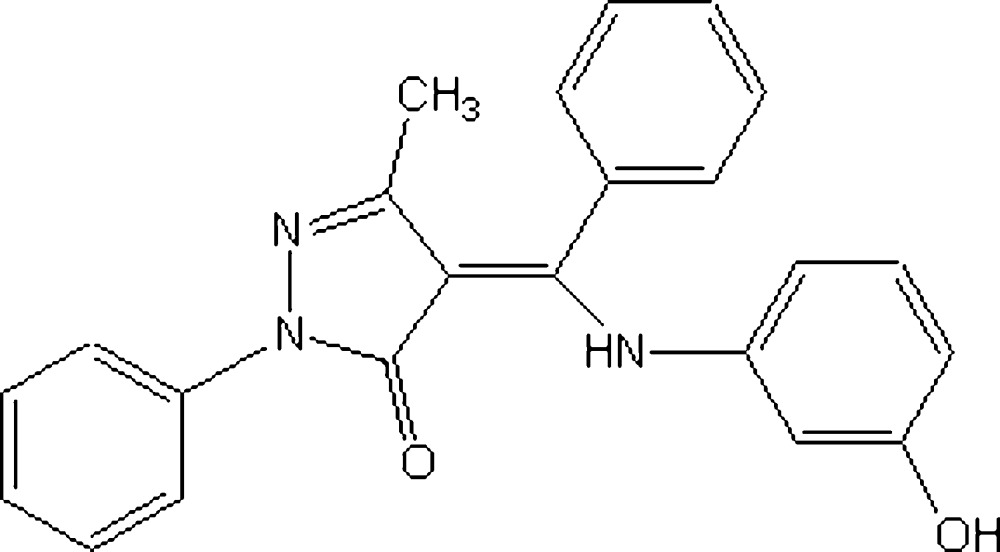


## Experimental

### 

#### Crystal data

C_23_H_19_N_3_O_2_
*M* *_r_* = 369.41
Triclinic, 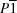

*a* = 9.5239 (3) Å
*b* = 10.4564 (4) Å
*c* = 10.8120 (4) Å
α = 66.870 (1)°
β = 72.208 (1)°
γ = 72.908 (1)°
*V* = 924.04 (6) Å^3^
*Z* = 2
Mo *K*α radiation
μ = 0.09 mm^−1^
*T* = 173 K
0.18 × 0.18 × 0.12 mm

#### Data collection

Bruker APEXII CCD diffractometer
Absorption correction: multi-scan (*SADABS*; Sheldrick, 1995 ▶) *T*_min_ = 0.985, *T*_max_ = 0.990
11733 measured reflections
4227 independent reflections
3385 reflections with *I* > 2σ(*I*)
*R*_int_ = 0.020

#### Refinement

*R*[*F* ^2^ > 2σ(*F* ^2^)] = 0.038
*wR*(*F* ^2^) = 0.096
*S* = 1.05
4227 reflections
262 parameters
H atoms treated by a mixture of independent and constrained refinement
Δρ_max_ = 0.27 e Å^−3^
Δρ_min_ = −0.19 e Å^−3^

### 

Data collection: *APEX2* (Bruker, 2006 ▶); cell refinement: *SAINT* (Bruker, 2006 ▶); data reduction: *SAINT*; program(s) used to solve structure: *SHELXS97* (Sheldrick, 2008 ▶); program(s) used to refine structure: *SHELXL97* (Sheldrick, 2008 ▶); molecular graphics: *ORTEPIII* (Burnett & Johnson, 1996 ▶), *ORTEP-3 for Windows* (Farrugia, 1997 ▶) and *PLATON* (Spek, 2009 ▶); software used to prepare material for publication: *publCIF* (Westrip, 2010 ▶).

## Supplementary Material

Crystal structure: contains datablock(s) I, global. DOI: 10.1107/S1600536812023082/vm2173sup1.cif

Structure factors: contains datablock(s) I. DOI: 10.1107/S1600536812023082/vm2173Isup2.hkl

Supplementary material file. DOI: 10.1107/S1600536812023082/vm2173Isup3.cml

Additional supplementary materials:  crystallographic information; 3D view; checkCIF report

## Figures and Tables

**Table 1:** Hydrogen-bond geometry (Å, °)

*D*—H⋯*A*	*D*—H	H⋯*A*	*D*⋯*A*	*D*—H⋯*A*
N1—H1*N*⋯O2	0.933 (16)	1.792 (17)	2.6189 (13)	146.1 (14)
O1—H1⋯N2^i^	0.93 (2)	1.84 (2)	2.7494 (13)	169.1 (18)

Symmetry code: (i) 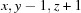.

**Fig. 1.**
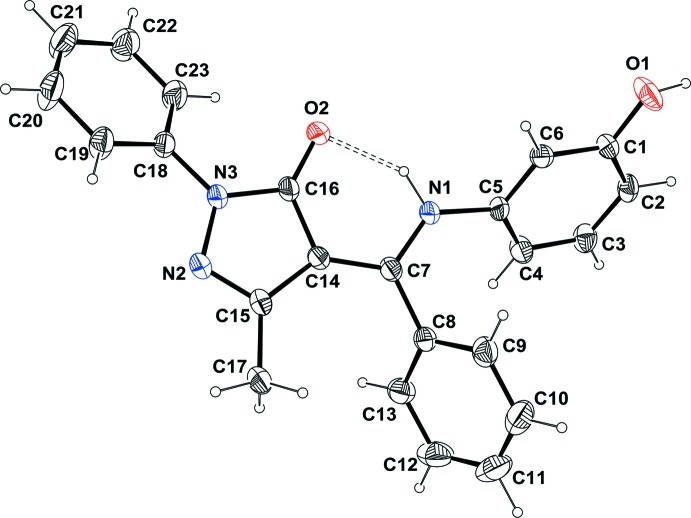
The asymmetric unit of (I) with the atom-labelling scheme. Displacement ellipsoids are drawn at the 50% probability level. H atoms are represented as small spheres of arbitrary radii. Hydrogen bond is shown as dashed line.

**Fig. 2.**
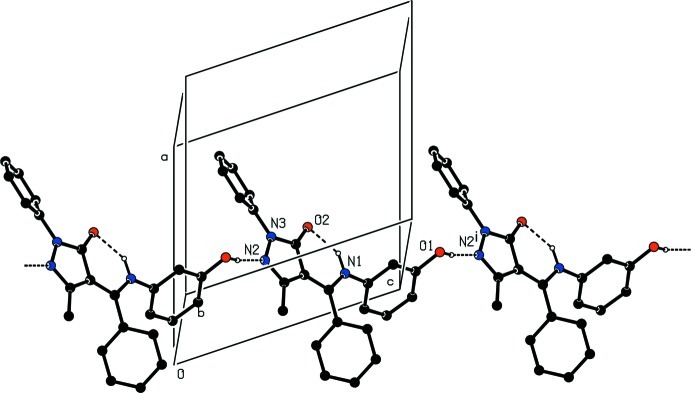
Partial packing view showing the intra and intermolecular hydrogen bonds (dashed lines). H atoms not involved in hydrogen bondings have been omitted for clarity [symmetry code: (i) x, y - 1, z + 1].

## supplementary crystallographic information

### Comment

4-Acyl-5-pyrazolones, a family of flexible β-diketonates and their derived Schiff bases are analgesics, antipyretics, anti-inflammatory agents and insecticides (Morris *et al.*, 1986; Metwally *et al.*, 1985; Casas *et al.*, 2007; Raja *et al.*, 2012). They have been widely used as extractants for metal traces (Delgado *et al.*, 2006; Liskovskaya *et al.*, 2006). Many of these compounds exhibit keto-enol tautomerism (Peng *et al.*, 2004).

The reaction of 1-phenyl-3-methyl-4-benzoyl-5-pyrazolone with primary amines affords Schiff bases that can function as N– and O-donor tridentates ligands,(Wang *et al.*, 2002; Ramasamy *et al.*, 2010; Thakar *et al.*, 2010; Xu *et al.*, 2006; Zhu *et al.*, 2005; Wang *et al.*, 2003). In order to expand this field, a novel Schiff base has been synthesized and its crystal structure is reported herein for the first time. The compound (I) was prepared from the reaction of 1-phenyl-3-methyl-4-benzoyl-5-pyrazolone (H1PMBP) and 3-aminophenol. The asymmetric unit of structure (I), and the atomic numbering used, are illustrated in Fig.1.

Steric hindrance affects this structure: the pyrazolone ring C14—N3 is not coplanar with the C18–C23 benzene ring and not perpendicular to the other two benzene rings C8–C13 and C1–C6. The dihedral angles are 30.92 (6), 60.96 (4) and 57.01 (4)°.

The O atom of the 3-methyl-1-phenylpyrazol-5-one unit and the N atom of the (3-hydroxyphenyl) amine group are available for coordination with metals. The pyrazolone ring is planar and atoms O2, C16, C14, C7 and N1 are coplanar, the largest deviation being 0.0179 (7) Å for atom C16. The dihedral angle between this plane (O2 C16 C14 C7 N1) and the pyrazolone ring of PMBP is 4.43 (8)°, close to the values of 4.01 (12)° found in (4*Z*)-[4 fluorobenzylamino)(phenyl)-methylene]-3-methyl-1-phenyl-1*H*-pyrazol-5(4*H*)-one and 5.05 (3)° found in 4-[(2-hydroxyphenylamino)phenylmethylene] -5-methyl-2-phenyl-2*H*-pyrazol-3(4*H*)-one, respectively (Xu *et al.*, 2006; Wang *et al.*, 2002).

The C15—N2—N3—C18 torsion angle is 176.73 (10)° , different from the value of 16.7 (3)° in 3-(2,3-dihydro-1,5-dimethyl-3-oxo-2-phenylpyrazol-4-ylmino)-4,4,4-trifluoro-1-(2-thienyl)-butane-1,2-dione (Wang *et al.*, 2002). Small torsion angles for N1 C7 C14 C16 [5.66 (17); O1 C1 C6 C5 [-178.76 (11)° ]and N1 C5 C6 C1 [-175.56 (11)° ] show that atoms O1, N1 and O2 are in a *cis* conformation and can act as the coordinating atoms of a tridentate ligand.

In the pyrazole ring, the bond lengths C14—C16, C14—C15, C15—N2, N2—N3 and C16—N3 lie between classical single and double bond lengths, indicating extensive conjugation and electron delocalization. The bond angles within this ring deviate by up to 4° from the 108° angle of a regular pentagon.

A strong intramolecular N1—H1N···O2 hydrogen bond (Table 1) is observed, leading to an enamine–keto tautomeric form. This case is similar to that found by Xu *et al.* (2006) for 4-[(2-hydroxyphenylamino)phenylmethylene] -5-methyl-2-phenyl-2*H*-pyrazol-3(4*H*)-one [N—O = 2.75 (3) Å and N3—H3···O1 = 143 (4)°]. The molecule is further stabilized by an intermolecular O—H···N hydrogen bond (Table1, Fig.2). Intermolecular O—H···N hydrogen bonds link molecules forming chains parallel to the [0 1 -1] direction. Part of the chain structure is shown in Fig.2.

### Experimental

All reagents were obtained from commercial sources and used without further purification. H1PMBP was synthesized according to the method proposed by Jensen (Jensen,1959). Ethanol solution of 139 mg (0.1 mol) of H1PMBP and 54.5 mg (0.1 mol) of *m*-aminophenol were refluxed together for 24 h over a steam bath. The excess solvent was removed by evaporation. The title compound separated out as a yellow powder, which was collected, dried in air and dissolved afterwards in a hot mixture ethanol/water (9.5/0.5). A bright yellow single crystals, suitable for X-ray analysis, were obtained by slow cooling of a warmed ethanol solution for one night. The product is stable in air, and soluble in acetone and ethanol. Elemental analysis: calculated C 74.78, H 5.18, N 11.37%; found C 74.34, H 5.20, N 11.33%.

### Refinement

The H atoms, except for the H-atoms of the OH and NH groups which were located from Fourier difference maps, were positioned geometrically and refined using a riding model, with C—H = 0.95–0.99 Å and with *U*_iso_(H) = 1.2 (1.5 for methyl groups) times *U*_eq_(C).

### Crystal data

**Table:**

C_23_H_19_N_3_O_2_	*Z* = 2
*M**_r_* = 369.41	*F*(000) = 388
Triclinic, *P*1	*D*_x_ = 1.328 Mg m^−^^3^
Hall symbol: -P 1	Mo *K*α radiation, λ = 0.71073 Å
*a* = 9.5239 (3) Å	Cell parameters from 5178 reflections
*b* = 10.4564 (4) Å	θ = 2.2–27.5°
*c* = 10.8120 (4) Å	µ = 0.09 mm^−^^1^
α = 66.870 (1)°	*T* = 173 K
β = 72.208 (1)°	Prism, yellow
γ = 72.908 (1)°	0.18 × 0.18 × 0.12 mm
*V* = 924.04 (6) Å^3^	

### Data collection

**Table:**

Bruker APEXII CCD diffractometer	4227 independent reflections
Radiation source: fine-focus sealed tube	3385 reflections with *I* > 2σ(*I*)
Graphite monochromator	*R*_int_ = 0.020
φ and ω scans	θ_max_ = 27.5°, θ_min_ = 2.1°
Absorption correction: multi-scan (*SADABS*; Sheldrick, 1995)	*h* = −12→12
*T*_min_ = 0.985, *T*_max_ = 0.990	*k* = −13→13
11733 measured reflections	*l* = −14→14

### Refinement

**Table:**

Refinement on *F*^2^	Primary atom site location: structure-invariant direct methods
Least-squares matrix: full	Secondary atom site location: difference Fourier map
*R*[*F*^2^ > 2σ(*F*^2^)] = 0.038	Hydrogen site location: inferred from neighbouring sites
*wR*(*F*^2^) = 0.096	H atoms treated by a mixture of independent and constrained refinement
*S* = 1.05	*w* = 1/[σ^2^(*F*_o_^2^) + (0.0452*P*)^2^ + 0.2126*P*] where *P* = (*F*_o_^2^ + 2*F*_c_^2^)/3
4227 reflections	(Δ/σ)_max_ < 0.001
262 parameters	Δρ_max_ = 0.27 e Å^−^^3^
0 restraints	Δρ_min_ = −0.19 e Å^−^^3^

### Special details

**Table:**

Geometry. All e.s.d.'s (except the e.s.d. in the dihedral angle between two l.s. planes) are estimated using the full covariance matrix. The cell e.s.d.'s are taken into account individually in the estimation of e.s.d.'s in distances, angles and torsion angles; correlations between e.s.d.'s in cell parameters are only used when they are defined by crystal symmetry. An approximate (isotropic) treatment of cell e.s.d.'s is used for estimating e.s.d.'s involving l.s. planes.
Refinement. Refinement of *F*^2^ against ALL reflections. The weighted *R*-factor *wR* and goodness of fit *S* are based on *F*^2^, conventional *R*-factors *R* are based on *F*, with *F* set to zero for negative *F*^2^. The threshold expression of *F*^2^ > σ(*F*^2^) is used only for calculating *R*-factors(gt) *etc*. and is not relevant to the choice of reflections for refinement. *R*-factors based on *F*^2^ are statistically about twice as large as those based on *F*, and *R*- factors based on ALL data will be even larger.

### Fractional atomic coordinates and isotropic or equivalent isotropic displacement parameters (Å^2^)

**Table:**

	*x*	*y*	*z*	*U*_iso_*/*U*_eq_	
C1	−0.01116 (13)	0.22740 (12)	1.05711 (11)	0.0238 (2)	
C2	−0.09952 (13)	0.14965 (12)	1.04593 (12)	0.0248 (2)	
H2	−0.1181	0.0632	1.1168	0.030*	
C3	−0.16031 (14)	0.19913 (13)	0.93055 (12)	0.0279 (3)	
H3	−0.2208	0.1457	0.9232	0.034*	
C4	−0.13494 (14)	0.32492 (13)	0.82548 (12)	0.0274 (3)	
H4	−0.1776	0.3579	0.7470	0.033*	
C5	−0.04596 (13)	0.40146 (12)	0.83745 (11)	0.0223 (2)	
C6	0.01657 (13)	0.35299 (12)	0.95185 (12)	0.0237 (2)	
H6	0.0785	0.4056	0.9584	0.028*	
C7	−0.08678 (13)	0.63561 (12)	0.65184 (11)	0.0219 (2)	
C8	−0.25221 (13)	0.65063 (12)	0.68941 (12)	0.0232 (2)	
C9	−0.33026 (14)	0.64999 (13)	0.82172 (13)	0.0300 (3)	
H9	−0.2779	0.6444	0.8863	0.036*	
C10	−0.48420 (16)	0.65755 (15)	0.85869 (16)	0.0407 (3)	
H10	−0.5378	0.6578	0.9485	0.049*	
C11	−0.56012 (16)	0.66473 (16)	0.76479 (18)	0.0455 (4)	
H11	−0.6657	0.6690	0.7908	0.055*	
C12	−0.48350 (16)	0.66578 (16)	0.63376 (17)	0.0419 (4)	
H12	−0.5364	0.6711	0.5697	0.050*	
C13	−0.32956 (14)	0.65906 (14)	0.59534 (14)	0.0304 (3)	
H13	−0.2769	0.6602	0.5049	0.036*	
C14	−0.00957 (13)	0.73156 (12)	0.53841 (11)	0.0222 (2)	
C15	−0.05592 (13)	0.86809 (12)	0.44249 (11)	0.0225 (2)	
C16	0.15253 (13)	0.70331 (12)	0.50200 (11)	0.0235 (2)	
C17	−0.20976 (14)	0.95569 (13)	0.43172 (13)	0.0307 (3)	
H17A	−0.2025	1.0550	0.3791	0.046*	
H17B	−0.2716	0.9486	0.5243	0.046*	
H17C	−0.2561	0.9210	0.3847	0.046*	
C18	0.33584 (13)	0.83449 (13)	0.30742 (11)	0.0240 (2)	
C19	0.36999 (15)	0.96867 (14)	0.24046 (13)	0.0316 (3)	
H19	0.2952	1.0506	0.2464	0.038*	
C20	0.51470 (17)	0.98159 (16)	0.16477 (16)	0.0442 (4)	
H20	0.5384	1.0733	0.1181	0.053*	
C21	0.62474 (17)	0.86396 (17)	0.15593 (16)	0.0449 (4)	
H21	0.7240	0.8742	0.1053	0.054*	
C22	0.58928 (16)	0.73101 (16)	0.22143 (15)	0.0410 (3)	
H22	0.6645	0.6495	0.2148	0.049*	
C23	0.44521 (15)	0.71527 (14)	0.29675 (14)	0.0337 (3)	
H23	0.4213	0.6235	0.3408	0.040*	
N1	−0.00339 (12)	0.52529 (11)	0.72968 (10)	0.0260 (2)	
N2	0.06091 (11)	0.91852 (10)	0.35404 (9)	0.0235 (2)	
N3	0.18928 (11)	0.81690 (10)	0.38753 (10)	0.0235 (2)	
O1	0.05168 (12)	0.18868 (11)	1.16665 (10)	0.0402 (3)	
O2	0.24418 (9)	0.60062 (9)	0.55996 (9)	0.0323 (2)	
H1N	0.0991 (19)	0.5271 (17)	0.6993 (17)	0.044 (4)*	
H1	0.049 (2)	0.096 (2)	1.223 (2)	0.068 (6)*	

### Atomic displacement parameters (Å^2^)

**Table:**

	*U*^11^	*U*^22^	*U*^33^	*U*^12^	*U*^13^	*U*^23^
C1	0.0256 (6)	0.0201 (6)	0.0209 (5)	−0.0036 (4)	−0.0070 (4)	−0.0011 (4)
C2	0.0292 (6)	0.0175 (5)	0.0218 (5)	−0.0074 (4)	−0.0011 (5)	−0.0020 (4)
C3	0.0337 (7)	0.0248 (6)	0.0267 (6)	−0.0127 (5)	−0.0049 (5)	−0.0063 (5)
C4	0.0338 (7)	0.0265 (6)	0.0220 (5)	−0.0103 (5)	−0.0089 (5)	−0.0029 (5)
C5	0.0211 (5)	0.0184 (5)	0.0205 (5)	−0.0049 (4)	−0.0029 (4)	0.0002 (4)
C6	0.0216 (6)	0.0197 (5)	0.0264 (6)	−0.0060 (4)	−0.0071 (4)	−0.0015 (5)
C7	0.0247 (6)	0.0216 (6)	0.0195 (5)	−0.0074 (4)	−0.0079 (4)	−0.0027 (4)
C8	0.0231 (6)	0.0173 (5)	0.0254 (5)	−0.0057 (4)	−0.0063 (4)	−0.0011 (4)
C9	0.0329 (7)	0.0233 (6)	0.0280 (6)	−0.0064 (5)	−0.0040 (5)	−0.0038 (5)
C10	0.0332 (7)	0.0286 (7)	0.0441 (8)	−0.0056 (6)	0.0080 (6)	−0.0082 (6)
C11	0.0214 (7)	0.0317 (7)	0.0719 (11)	−0.0054 (5)	−0.0030 (7)	−0.0106 (7)
C12	0.0309 (7)	0.0354 (8)	0.0619 (9)	−0.0061 (6)	−0.0225 (7)	−0.0097 (7)
C13	0.0284 (6)	0.0288 (7)	0.0331 (6)	−0.0067 (5)	−0.0109 (5)	−0.0054 (5)
C14	0.0227 (6)	0.0219 (6)	0.0202 (5)	−0.0063 (4)	−0.0081 (4)	−0.0013 (4)
C15	0.0272 (6)	0.0213 (6)	0.0174 (5)	−0.0055 (4)	−0.0061 (4)	−0.0032 (4)
C16	0.0250 (6)	0.0223 (6)	0.0214 (5)	−0.0101 (5)	−0.0081 (4)	0.0010 (4)
C17	0.0290 (6)	0.0259 (6)	0.0251 (6)	−0.0005 (5)	−0.0058 (5)	0.0004 (5)
C18	0.0256 (6)	0.0255 (6)	0.0200 (5)	−0.0107 (5)	−0.0045 (4)	−0.0030 (5)
C19	0.0327 (7)	0.0236 (6)	0.0322 (6)	−0.0102 (5)	0.0014 (5)	−0.0059 (5)
C20	0.0408 (8)	0.0318 (7)	0.0482 (8)	−0.0192 (6)	0.0101 (6)	−0.0073 (6)
C21	0.0311 (7)	0.0428 (8)	0.0477 (8)	−0.0141 (6)	0.0095 (6)	−0.0104 (7)
C22	0.0320 (7)	0.0336 (8)	0.0440 (8)	−0.0035 (6)	0.0012 (6)	−0.0086 (6)
C23	0.0326 (7)	0.0248 (6)	0.0362 (7)	−0.0092 (5)	−0.0040 (5)	−0.0025 (5)
N1	0.0220 (5)	0.0240 (5)	0.0247 (5)	−0.0094 (4)	−0.0078 (4)	0.0050 (4)
N2	0.0269 (5)	0.0198 (5)	0.0199 (4)	−0.0046 (4)	−0.0070 (4)	−0.0011 (4)
N3	0.0236 (5)	0.0200 (5)	0.0222 (5)	−0.0077 (4)	−0.0071 (4)	0.0017 (4)
O1	0.0596 (7)	0.0281 (5)	0.0339 (5)	−0.0194 (5)	−0.0282 (5)	0.0099 (4)
O2	0.0235 (4)	0.0282 (5)	0.0338 (5)	−0.0087 (4)	−0.0119 (4)	0.0084 (4)

### Geometric parameters (Å, º)

**Table:**

C1—O1	1.3560 (14)	C13—H13	0.9500
C1—C2	1.3875 (17)	C14—C15	1.4334 (15)
C1—C6	1.3913 (15)	C14—C16	1.4413 (16)
C2—C3	1.3847 (17)	C15—N2	1.3150 (15)
C2—H2	0.9500	C15—C17	1.4936 (16)
C3—C4	1.3872 (16)	C16—O2	1.2499 (14)
C3—H3	0.9500	C16—N3	1.3747 (14)
C4—C5	1.3865 (17)	C17—H17A	0.9800
C4—H4	0.9500	C17—H17B	0.9800
C5—C6	1.3863 (16)	C17—H17C	0.9800
C5—N1	1.4259 (14)	C18—C19	1.3874 (17)
C6—H6	0.9500	C18—C23	1.3887 (18)
C7—N1	1.3329 (15)	C18—N3	1.4211 (15)
C7—C14	1.4026 (15)	C19—C20	1.3859 (18)
C7—C8	1.4784 (16)	C19—H19	0.9500
C8—C13	1.3907 (17)	C20—C21	1.376 (2)
C8—C9	1.3945 (17)	C20—H20	0.9500
C9—C10	1.3835 (19)	C21—C22	1.380 (2)
C9—H9	0.9500	C21—H21	0.9500
C10—C11	1.384 (2)	C22—C23	1.3847 (18)
C10—H10	0.9500	C22—H22	0.9500
C11—C12	1.378 (2)	C23—H23	0.9500
C11—H11	0.9500	N1—H1N	0.933 (16)
C12—C13	1.3846 (18)	N2—N3	1.3998 (13)
C12—H12	0.9500	O1—H1	0.93 (2)
			
O1—C1—C2	123.78 (10)	C7—C14—C16	120.73 (10)
O1—C1—C6	116.53 (11)	C15—C14—C16	105.31 (9)
C2—C1—C6	119.69 (10)	N2—C15—C14	111.05 (10)
C3—C2—C1	119.42 (11)	N2—C15—C17	118.70 (10)
C3—C2—H2	120.3	C14—C15—C17	130.25 (10)
C1—C2—H2	120.3	O2—C16—N3	125.58 (11)
C2—C3—C4	121.52 (11)	O2—C16—C14	129.22 (10)
C2—C3—H3	119.2	N3—C16—C14	105.19 (10)
C4—C3—H3	119.2	C15—C17—H17A	109.5
C5—C4—C3	118.58 (11)	C15—C17—H17B	109.5
C5—C4—H4	120.7	H17A—C17—H17B	109.5
C3—C4—H4	120.7	C15—C17—H17C	109.5
C6—C5—C4	120.65 (10)	H17A—C17—H17C	109.5
C6—C5—N1	116.68 (10)	H17B—C17—H17C	109.5
C4—C5—N1	122.45 (10)	C19—C18—C23	120.14 (11)
C5—C6—C1	120.13 (11)	C19—C18—N3	120.74 (11)
C5—C6—H6	119.9	C23—C18—N3	119.12 (11)
C1—C6—H6	119.9	C20—C19—C18	119.12 (13)
N1—C7—C14	116.75 (10)	C20—C19—H19	120.4
N1—C7—C8	119.11 (10)	C18—C19—H19	120.4
C14—C7—C8	124.14 (10)	C21—C20—C19	121.20 (13)
C13—C8—C9	119.82 (11)	C21—C20—H20	119.4
C13—C8—C7	120.26 (11)	C19—C20—H20	119.4
C9—C8—C7	119.86 (11)	C20—C21—C22	119.28 (13)
C10—C9—C8	119.79 (13)	C20—C21—H21	120.4
C10—C9—H9	120.1	C22—C21—H21	120.4
C8—C9—H9	120.1	C21—C22—C23	120.66 (14)
C9—C10—C11	120.01 (14)	C21—C22—H22	119.7
C9—C10—H10	120.0	C23—C22—H22	119.7
C11—C10—H10	120.0	C22—C23—C18	119.59 (12)
C12—C11—C10	120.40 (13)	C22—C23—H23	120.2
C12—C11—H11	119.8	C18—C23—H23	120.2
C10—C11—H11	119.8	C7—N1—C5	129.53 (10)
C11—C12—C13	120.10 (14)	C7—N1—H1N	112.5 (10)
C11—C12—H12	119.9	C5—N1—H1N	117.4 (10)
C13—C12—H12	119.9	C15—N2—N3	106.92 (9)
C12—C13—C8	119.87 (13)	C16—N3—N2	111.43 (9)
C12—C13—H13	120.1	C16—N3—C18	126.83 (10)
C8—C13—H13	120.1	N2—N3—C18	121.73 (9)
C7—C14—C15	133.89 (11)	C1—O1—H1	112.6 (12)
			
O1—C1—C2—C3	179.16 (12)	C16—C14—C15—C17	−177.58 (12)
C6—C1—C2—C3	−0.70 (18)	C7—C14—C16—O2	−1.3 (2)
C1—C2—C3—C4	0.02 (19)	C15—C14—C16—O2	176.13 (13)
C2—C3—C4—C5	0.24 (19)	C7—C14—C16—N3	179.66 (11)
C3—C4—C5—C6	0.17 (19)	C15—C14—C16—N3	−2.87 (13)
C3—C4—C5—N1	174.57 (11)	C23—C18—C19—C20	−0.9 (2)
C4—C5—C6—C1	−0.85 (18)	N3—C18—C19—C20	179.14 (12)
N1—C5—C6—C1	−175.56 (11)	C18—C19—C20—C21	−0.5 (2)
O1—C1—C6—C5	−178.76 (11)	C19—C20—C21—C22	1.3 (3)
C2—C1—C6—C5	1.12 (18)	C20—C21—C22—C23	−0.8 (3)
N1—C7—C8—C13	−123.11 (13)	C21—C22—C23—C18	−0.6 (2)
C14—C7—C8—C13	57.38 (17)	C19—C18—C23—C22	1.4 (2)
N1—C7—C8—C9	54.03 (16)	N3—C18—C23—C22	−178.59 (12)
C14—C7—C8—C9	−125.48 (13)	C14—C7—N1—C5	−169.85 (12)
C13—C8—C9—C10	0.09 (18)	C8—C7—N1—C5	10.60 (19)
C7—C8—C9—C10	−177.06 (11)	C6—C5—N1—C7	−140.00 (13)
C8—C9—C10—C11	0.4 (2)	C4—C5—N1—C7	45.39 (19)
C9—C10—C11—C12	−0.6 (2)	C14—C15—N2—N3	0.31 (13)
C10—C11—C12—C13	0.3 (2)	C17—C15—N2—N3	179.62 (10)
C11—C12—C13—C8	0.3 (2)	O2—C16—N3—N2	−175.82 (12)
C9—C8—C13—C12	−0.45 (19)	C14—C16—N3—N2	3.24 (13)
C7—C8—C13—C12	176.69 (12)	O2—C16—N3—C18	5.2 (2)
N1—C7—C14—C15	−170.95 (13)	C14—C16—N3—C18	−175.73 (11)
C8—C7—C14—C15	8.6 (2)	C15—N2—N3—C16	−2.30 (13)
N1—C7—C14—C16	5.66 (17)	C15—N2—N3—C18	176.73 (10)
C8—C7—C14—C16	−174.82 (11)	C19—C18—N3—C16	−149.41 (12)
C7—C14—C15—N2	178.60 (13)	C23—C18—N3—C16	30.60 (18)
C16—C14—C15—N2	1.63 (13)	C19—C18—N3—N2	31.72 (17)
C7—C14—C15—C17	−0.6 (2)	C23—C18—N3—N2	−148.27 (12)

### Hydrogen-bond geometry (Å, º)

**Table:**

*D*—H···*A*	*D*—H	H···*A*	*D*···*A*	*D*—H···*A*
N1—H1*N*···O2	0.933 (16)	1.792 (17)	2.6189 (13)	146.1 (14)
O1—H1···N2^i^	0.93 (2)	1.84 (2)	2.7494 (13)	169.1 (18)

Symmetry code: (i) *x*, *y*−1, *z*+1.
